# Association of winter exposure with ischemic stroke risk in nonvalvular atrial fibrillation within a CHADS₂-based risk framework: a nationwide Japanese administrative claims database study

**DOI:** 10.1186/s43044-026-00766-z

**Published:** 2026-07-16

**Authors:** Ryusuke Sakuma, Shuko Nojiri, Takuya Uematsu, Masashi Nagao, Muneaki Ishijima, Yuji Nishizaki

**Affiliations:** 1 Clinical Translational Science, Juntendo University School of Medicine, Tokyo, Japan; 2 Medical Technology Innovation Center, Juntendo University, Tokyo, Japan; 3 Department of Hospital Pharmacy, Juntendo University Shizuoka Hospital, Shizuoka, Japan; 4 Department of Medicine for Orthopaedics and Motor Organ, Juntendo University Graduate School of Medicine, Tokyo, Japan; 5 Division of Medical Education, Juntendo University School of Medicine, Tokyo, Japan

**Keywords:** Nonvalvular atrial fibrillation, Ischemic stroke, CHADS₂ score, Seasonal variation, Winter exposure

## Abstract

**Background:**

Ischemic stroke, including transient ischemic attack, exhibits seasonal variation, and the association between winter exposure and ischemic stroke risk in patients with nonvalvular atrial fibrillation (NVAF) has not been fully characterized at the population level. We investigated the association of winter exposure with ischemic stroke risk in Japanese patients with NVAF within a CHADS₂-based risk framework.

**Results:**

We analyzed nationwide Japanese insurance claims data (2014–2022) to identify patients ≥ 60 years with newly diagnosed NVAF and subsequently conducted Poisson regression analyses. The outcome was hospitalization for ischemic stroke. Among 389,314 patients (median age 80 years), 24,576 (6.3%) had ischemic stroke. The incidence rate peaked in winter (December–February), reaching 35.0 per 1,000 person-years, the highest among all seasons. In a Poisson regression model adjusted for CHADS₂ components, with winter treated as a binary exposure, winter remained independently associated with ischemic stroke risk (adjusted incidence rate ratio 1.06, 95% CI 1.03–1.09). Adding winter exposure to the CHADS₂ framework improved model fit but resulted in only negligible improvement in discrimination (AUC 0.690 vs. 0.691). In interaction analyses with CHADS₂ components, the association between winter exposure and ischemic stroke risk was stronger in patients without prior ischemic stroke than in those with prior ischemic stroke (*p* = 0.033).

**Conclusions:**

In patients with NVAF, winter exposure was associated with a modest increase in ischemic stroke risk at the population level. The stronger association observed among patients without prior ischemic stroke may support seasonal risk awareness and population-level risk communication in the primary prevention setting.

**Supplementary Information:**

The online version contains supplementary material available at 10.1186/s43044-026-00766-z.

## Background

Atrial fibrillation (AF) is the most common sustained arrhythmia and a major risk factor for ischemic stroke and other cardiovascular complications [1, 2]. Globally, AF affects over 60 million people, and its prevalence increases with advancing age and the presence of comorbid cardiovascular disease [1, 2]. Nonvalvular atrial fibrillation (NVAF), the predominant subtype of AF, accounts for approximately 20–30% of ischemic stroke cases. The Framingham study showed that patients with untreated NVAF have an approximately five-fold higher risk of ischemic stroke than does the general population [2, 3].

The CHADS₂ score has long served as a cornerstone for risk stratification of ischemic stroke in NVAF and for guiding anticoagulant therapy. While CHADS₂ is simple and widely used in clinical practice, it does not incorporate potentially important environmental or temporal factors that may influence ischemic stroke risk [4]. Although several alternative risk models have been proposed, CHADS₂ remains the most clinically practical tool, balancing simplicity with an acceptable predictive accuracy [2, 5].

In addition to patient-related factors, seasonal variation influences cardiovascular events, including ischemic stroke. Registry studies from Denmark, New Zealand, Taiwan, and Japan have consistently reported a seasonally higher incidence of ischemic stroke in winter among patients with AF [6–8]. Nevertheless, the CHADS₂ score does not account for seasonal factors. Accordingly, we investigated the association between winter exposure and ischemic stroke risk in Japanese patients with NVAF after accounting for the established components of the CHADS₂ score.

## Methods

### Data source

This study utilized an anonymized health insurance claims database provided by DeSC Healthcare, Inc. (Tokyo, Japan). The structure and representativeness of this database have been described in detail in previous reports [9]. The database includes information on beneficiaries covered by employee-based health insurance, National Health Insurance, and the Late-Stage Elderly Healthcare System. In addition to standard medical claims data, it contains outpatient visit records, hospitalization information, Diagnosis Procedure Combination data, and health checkup records, including specific health checkups.

The observation period was from April 2014 to October 2022, and individuals who were enrolled in any insurance scheme for at least one month during this period were included. Medical records covering the full duration of each individual’s insurance coverage were available, enabling longitudinal follow-up. Data were available for 6,833,165 individuals [9].

### Study population

Patients newly diagnosed with AF between April 2014 and October 2022 were identified. Diagnosis was based on ICD-10 code I48.x [10]. The index date (baseline) was defined as the date of the first documented AF diagnosis. Patients were eligible if their index date fell within the observation period and they could be followed for at least 1 day; those whose diagnosis and end of follow-up occurred on the same day were excluded.

Follow-up extended until the first occurrence of any of the following events: (1) hospitalization for ischemic stroke; (2) end of the observation period (October 2022); or (3) the final documented medical encounter. Exclusion criteria were as follows: (1) rheumatic or valvular AF (ICD-10 I05.0–I05.2, I05.8–I05.9) without documented use of direct oral anticoagulants (DOACs), (2) history of mechanical valve replacement, or (3) a documented AF diagnosis before March 2014. Detailed definitions and code lists are provided in Additional file 1.

### Outcome

The primary outcome was the first hospitalization for ischemic stroke, including transient ischemic attack (TIA), occurring after the diagnosis of AF.

Ischemic stroke was defined as hospitalization with ICD-10 code I63.x [10], where the diagnosis and admission occurred on the same day or within 1 day.

TIA was defined as hospitalization with ICD-10 codes H34.0, G45.0, G45.1, G45.8, and G45.9 (excluding G45.4) [10], with diagnosis and admission required to occur on the same day or within 1 day.

H34.0 (amaurosis fugax) was clinically classified as an ocular ischemic attack and therefore included in the TIA definition.

Only the first qualifying event after AF diagnosis was included in the analysis.

### Exposure and covariates

The exposure variable was season, categorized into four groups according to calendar months: spring (March–May), summer (June–August), autumn (September–November), and winter (December–February). As ischemic stroke incidence peaked during winter, the season was modeled as a binary exposure variable, coded as 1 for winter (December–February) and 0 for all other months. The five components of the CHADS₂ score [4] were included as covariates: age ≥ 75 years, hypertension, diabetes, history of ischemic stroke, including TIA, and heart failure. History of ischemic stroke, including TIA, was identified from diagnosis records before the index AF diagnosis. Heart failure was defined as hospitalization with a principal diagnosis of ICD-10 I50.x or I11.0 within 3 months before the index AF diagnosis. Hypertension and diabetes were identified only when both diagnostic and pharmacologic criteria were met. Hypertension was defined as an ICD-10 code of I10–I15 accompanied by a prescription for antihypertensive medication (ATC C02, C03, C07, C08, or C09), and diabetes as an ICD-10 code of E10–E14 accompanied by a prescription for antidiabetic medication (ATC A10A or A10B). All covariates were treated as time-fixed variables and assigned to each monthly observation unit. ICD-10 codes and medication code definitions are summarized in Additional file 2; complete local disease codes corresponding to the ICD-10 definitions are provided in Additional file 3. Although the dataset also contained information on sex, DOAC use, and region code, these variables were not included in the primary multivariable model, as the analysis focused specifically on evaluating the association between winter exposure and ischemic stroke risk within a CHADS₂-based risk framework.

### Statistical analysis

The follow-up duration of each patient was partitioned into monthly intervals, each assigned an event indicator (event = 1/0) and corresponding person-time (1/12 person-year). Seasonal incidence rates (IR) of ischemic stroke were then calculated per 1,000 person-years.

To evaluate seasonal differences, incidence rate ratios (IRRs) and 95% confidence intervals (CIs) were estimated using univariate Poisson regression with winter as the reference category.

In the multivariable Poisson regression models, winter exposure was included in addition to all CHADS₂ components to evaluate its independent association with ischemic stroke risk and its contribution to seasonal variation in stroke incidence. Interaction terms between winter exposure and each CHADS₂ component were introduced to determine whether the association between winter exposure and ischemic stroke risk differed across risk factor subgroups.

Two sensitivity analyses were performed. First, the primary multivariable model was additionally adjusted for sex, DOAC use, geographic region, and calendar year. Second, the outcome definition was restricted to ischemic stroke, excluding TIA. The results of these analyses are presented in Additional file 4.

All analyses were conducted using R version 4.5.0 (R Foundation for Statistical Computing, Vienna, Austria). Statistical significance was set at a two-sided *p* < 0.05. Incidence rates and IRRs were reported with 95% CIs. Model variance was evaluated using Pearson χ²/df and deviance/df, and no evidence of substantial overdispersion was observed.

## Results

### Study population and patient disposition

Between April 2014 and October 2022, a total of 389,999 patients were newly diagnosed with AF. The following were excluded: 206 patients with no follow-up, 266 with a history of mechanical valve replacement, and 213 with rheumatic mitral valve disease but no record of direct oral anticoagulant (DOAC) use. Consequently, the final analysis included 389,314 patients with NVAF.

**Fig. 1:**
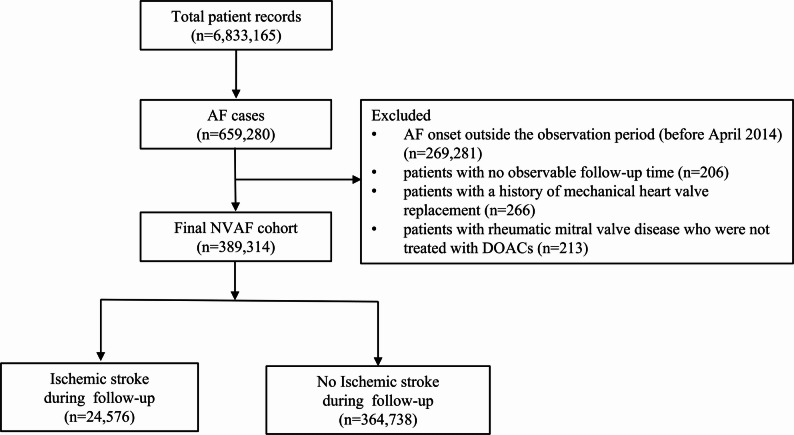
Patient disposition. Flow diagram of patient selection. Of 6,833,165 total patient records, 389,314 patients with newly diagnosed nonvalvular atrial fibrillation were included in the final analysis. During follow-up, 24,576 outcome events, including ischemic stroke or transient ischemic attack, occurred

Ischemic stroke occurred in 24,576 patients (6.3%), whereas 364,738 patients (93.7%) remained event-free during follow-up (Fig. 1).

### Baseline characteristics

The median age of the enrolled participants was 80 years (interquartile range [IQR], 75–86), and 50.9% were male. The median follow-up period was 1.5 years (IQR, 0.5–2.8), and the median CHADS₂ score was 2 (IQR, 1–3). The prevalences of major comorbidities were as follows: hypertension, 72.8%; diabetes, 20.4%; heart failure hospitalization, 11.9%; and prior ischemic stroke, 15.5%. DOACs were prescribed in 55.8% of the cohort. Compared with patients without ischemic stroke, those who developed stroke (*n* = 24,576) were generally older (median, 82 vs. 80 years) and had higher CHADS₂ scores (median, 3 vs. 2). They also had higher rates of hypertension (75.7% vs. 72.6%), diabetes (23.1% vs. 20.2%), and prior ischemic stroke (43.9% vs. 13.6%) (Table 1).

**Table 1:** Baseline characteristics of NVAF patients with and without ischemic stroke

Variable	Overall (*n* = 389314)	No ischemic stroke (*n* = 364738)	Ischemic stroke (*n* = 24576)
Age (years), median [IQR]	80.0 (75.0–86.0)	80.0 (74.0–86.0)	82.0 (77.0–87.0)
Follow-up duration (years), median [IQR]	1.5 (0.5–2.8)	1.5 (0.6–2.9)	0.6 (0.1–1.6)
CHADS_2_ score, median [IQR]	2.0 (1.0–3.0)	2.0 (1.0–3.0)	3.0 (2.0–4.0)
Age ≥ 75 years, n (%)	293,475 (75.4%)	272,870 (74.8%)	20,605 (83.8%)
Hypertension, n (%)	283,227 (72.8%)	264,624 (72.6%)	18,603 (75.7%)
Diabetes, n (%)	79,239 (20.4%)	73,564 (20.2%)	5675 (23.1%)
Heart failure hospitalization, n (%)	46,176 (11.9%)	43,328 (11.9%)	2848 (11.6%)
Prior ischemic stroke, n (%)	60,495 (15.5%)	49,717 (13.6%)	10,778 (43.9%)
DOAC use, n (%)	217,106 (55.8%)	201,618 (55.3%)	15,488 (63.0%)
Male sex, n (%)	198,081 (50.9%)	185,200 (50.8%)	12,881 (52.4%)

Values are shown as median [IQR] or n (%). In this table, ischemic stroke includes transient ischemic attack. Heart failure hospitalization was assessed within 3 months prior to the index date, and DOAC use was defined at AF diagnosis. Abbreviations: IQR, interquartile range; DOAC, direct oral anticoagulant

### Seasonal incidence of ischemic stroke

**Fig. 2:**
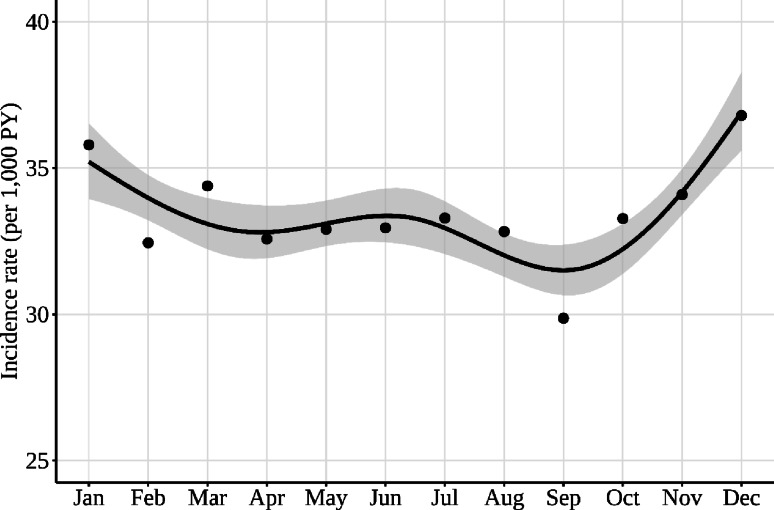
Monthly incidence of ischemic stroke (spline-smoothed). Spline-smoothed incidence rates (IRs) per 1,000 person-years with 95% confidence intervals (CIs) are shown by calendar month (April 2014–October 2022). Black points denote the observed monthly IRs, and the black line represents the fitted spline with 95% CI. Winter exposure in the primary analysis was defined as December through February, corresponding to the winter category used in the regression analyses

The monthly incidence of ischemic stroke increased from September through December, peaked in December, and remained elevated in January before declining from March onward and staying relatively low through September. Monthly event counts, person-years, and incidence rates are provided in Additional file 5. This trend was consistently observed throughout the study period (Fig. 2).

Seasonal analysis showed that the IR peaked in winter and was significantly lower in spring, summer, and autumn. In univariate Poisson regression, the IRR was 0.95 (95% CI 0.92–0.99, *p* = 0.008) in spring, 0.94 (95% CI 0.91–0.98, *p* = 0.001) in summer, and 0.92 (95% CI 0.89–0.96, *p* < 0.001) in autumn (Table 2).

**Table 2:** Seasonal incidence rates and IRRs of ischemic stroke (winter as reference)

Season (months)	Ischemic stroke	Person-years	IR (per 1,000 PY)	IRR vs. winter (reference)	95% CI	*p*-value
Winter (Dec–Feb)	6773	193561.9	35.0	1.00 (ref)	–	–
Spring (Mar–May)	5891	176514.3	33.4	0.95	(0.92–0.99)	0.0079
Summer (Jun–Aug)	5867	177644.9	33.0	0.94	(0.91–0.98)	0.0012
Autumn (Sep–Nov)	6045	186404.9	32.4	0.92	(0.89–0.96)	< 0.001

Crude incidence rates (IRs) per 1,000 person-years and IRRs (95% CIs) were estimated using univariate Poisson models with winter (December–February) as the reference. PY, person-years; CI, confidence interval; IRR, incidence rate ratio

### Clinical risk factors and seasonal effect

In univariate analysis, all CHADS₂ score components and winter exposure were significantly associated with ischemic stroke risk. The strongest association was observed for prior ischemic stroke (IRR 6.31, 95% CI 6.15–6.47), followed by age ≥ 75 years (IRR 2.08, 95% CI 2.04–2.13). Diabetes mellitus (IRR 1.34), hypertension (IRR 1.33), and hospitalization for heart failure (IRR 1.22) were significantly associated with increased risk. In addition, winter exposure was associated with a modest yet statistically significant increase in risk (IRR 1.06, 95% CI 1.03–1.09).

**Fig. 3:**
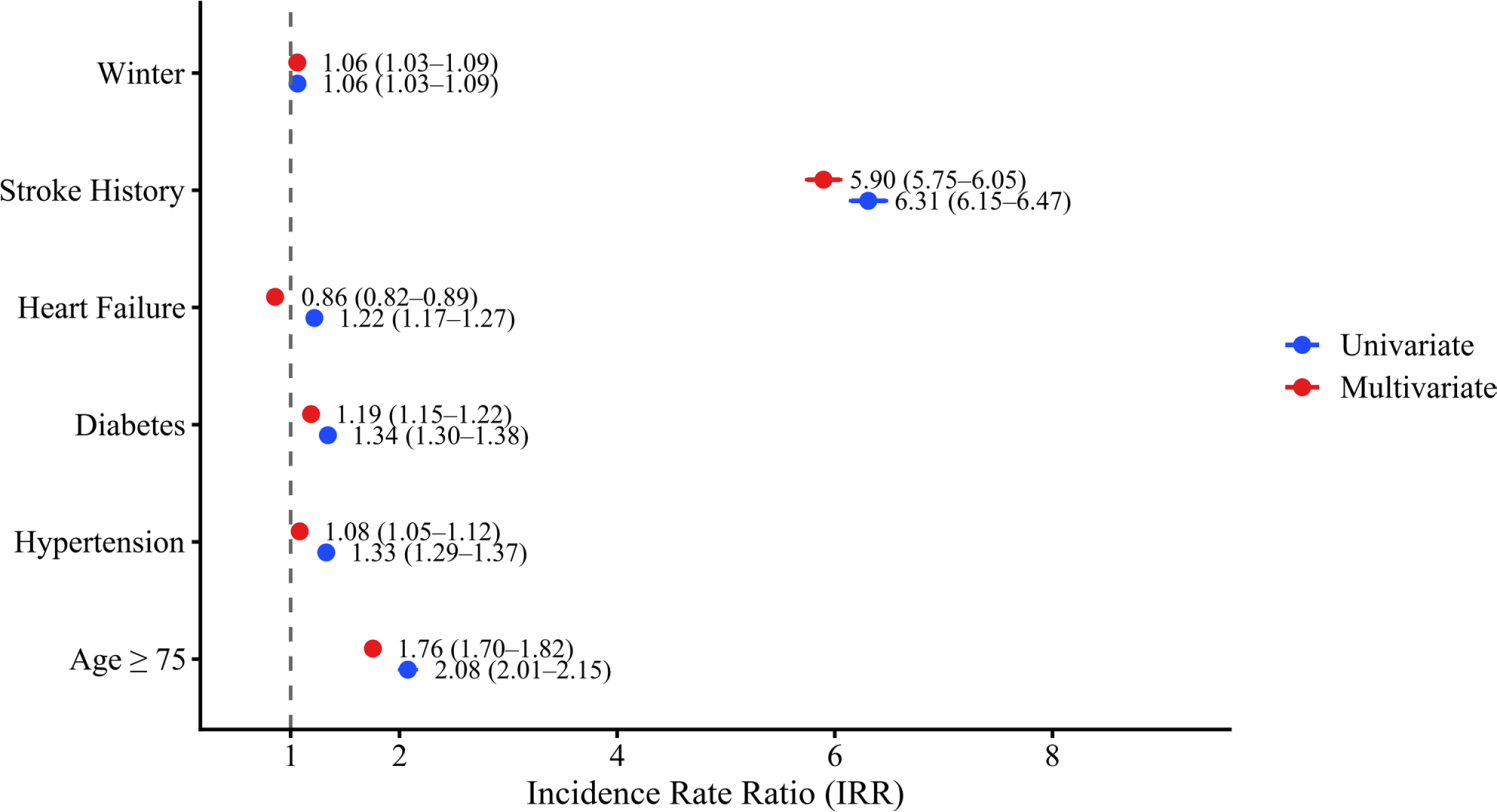
Incidence rate ratios (IRRs) by CHADS₂ score components and winter exposure. Univariate (blue) and multivariable (red) Poisson regression models are shown. Points denote IRRs with 95% confidence intervals (CIs) for winter exposure and for each CHADS₂ component. Stroke history indicates a prior history of ischemic stroke, including transient ischemic attack

These associations persisted in the multivariable model, with prior ischemic stroke (IRR 5.90) and age ≥ 75 years (IRR 1.76) being the most influential factors. Winter exposure also remained independently associated with ischemic stroke risk after adjustment (IRR 1.06, 95% CI 1.03–1.09; Fig. 3).

Adding winter exposure to the CHADS₂ score improved model fit (ΔAIC = − 14.1, likelihood ratio test *p* < 0.001, Table 3) but resulted in only a negligible increase in discriminatory power (AUC 0.690 vs. 0.691, Additional file 6).

**Table 3:** Comparison of model fit between CHADS₂-only and CHADS₂ plus winter exposure models

Model	AIC
CHADS₂ only	325470.1
CHADS₂ + winter exposure	325456.0
ΔAIC	−14.1

Lower AIC values indicate better model fit

Likelihood ratio test, χ²(1) = 16.07, *p* < 0.001

Of the 24,576 outcome events, 929 (3.8%) were classified as TIA events. In sensitivity analyses, the association between winter exposure and ischemic stroke risk remained materially unchanged after additional adjustment for sex, DOAC use, geographic region, and calendar year (IRR 1.08, 95% CI 1.05–1.12) and after exclusion of TIA events from the outcome definition (IRR 1.08, 95% CI 1.05–1.11; Additional file 4).

**Fig. 4:**
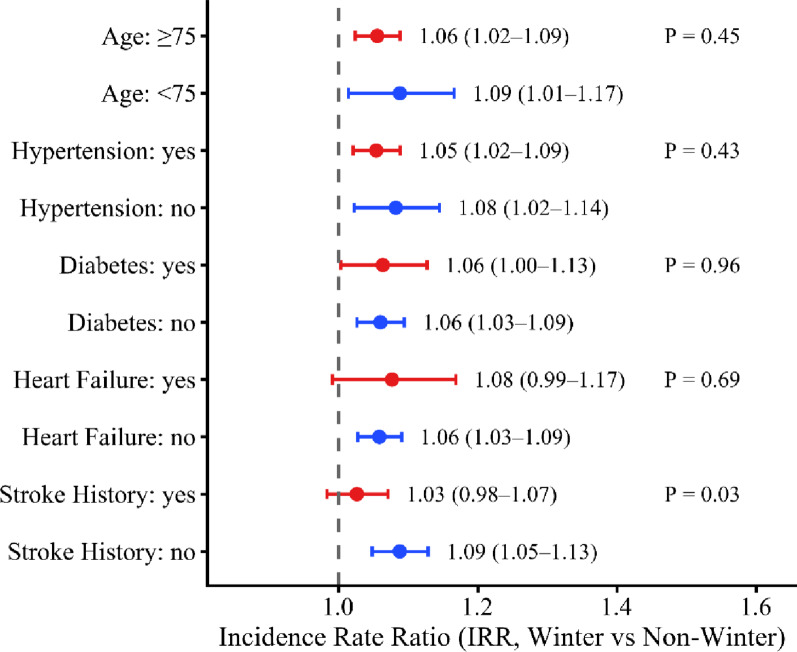
Stratified IRRs for winter versus non-winter by CHADS₂ score components. IRRs with 95% CIs are presented for winter versus non-winter within each CHADS₂ component. P values for interaction were obtained from multivariable Poisson regression analyses. Stroke history indicates a prior history of ischemic stroke, including transient ischemic attack. Heart failure indicates heart failure–related hospitalization within 3 months before the index date

Interaction analysis showed that the association between winter exposure and ischemic stroke risk was significantly stronger in patients without prior ischemic stroke than in those with prior ischemic stroke (interaction *p* = 0.033). No significant interactions were observed with other CHADS₂ components (all *p* > 0.4; Fig. 4).

## Discussion

This nationwide cohort study evaluated the association between winter exposure and ischemic stroke risk among patients with NVAF in Japan. Winter exposure was independently associated with ischemic stroke risk after adjustment for CHADS₂ components, with the incidence rate peaking during winter.

Although the magnitude of the association was modest, this association was consistent and robust across analyses. These findings support the interpretation of winter exposure as a population-level seasonal modifier of ischemic stroke risk in NVAF, rather than a major determinant of individual susceptibility. Notably, the association was more pronounced in patients without a prior history of ischemic stroke than in those with a previous event. This finding may suggest greater susceptibility to seasonal risk in the primary prevention setting than in the secondary prevention setting.

Collectively, these findings may help inform seasonal risk awareness and population-level risk communication.

This pattern aligns with prior reports demonstrating increased wintertime stroke incidence among patients with AF. Large-scale registry studies from Denmark and New Zealand [6], a nationwide claims-based cohort study in Taiwan [7], and a Japanese hospital-based registry (SHINKEN study) [8] have all consistently demonstrated similar seasonal patterns. This study extends these findings by analyzing a larger, nationally representative NVAF population using clearly defined criteria. However, the effect size observed in this study was smaller than that reported in some previous studies [6–8]. This difference may reflect the variation in age distribution, anticoagulant use [11–13], underlying cardiovascular risk, healthcare access, and analytic units (person-years calculated on a monthly basis using exact follow-up periods). Nevertheless, this study supports the interpretation of winter exposure as a modest population-level modifier of ischemic stroke risk.

The mechanism underlying the increased ischemic stroke risk in winter is considered multifactorial and biologically plausible. Epidemiological studies suggest that cold exposure activates the sympathetic nervous system, leading to vasoconstriction, increased blood pressure, and a procoagulant shift via elevated catecholamine levels [14, 15]. Reduced physical activity and increased time spent indoors may also lower venous return, thereby promoting left atrial stasis and thrombus formation. Furthermore, seasonal increases in fibrinogen and blood viscosity have been observed, supporting a heightened thrombotic tendency during winter [16, 17]. In regions with marked climatic variability, these physiological and behavioral factors may synergistically contribute to a pronounced seasonal pattern of embolic events [12, 18, 19].

From the perspective of predictive modeling, adding a winter indicator to the CHADS₂ score improved model fit (AIC) but resulted in only negligible improvement in discrimination (AUC). Because this study analyzed person-time at the monthly level rather than individual-level risk profiles, the winter indicator primarily captured temporal variation in stroke incidence across the population and therefore contributed minimally to individual-level risk discrimination. Accordingly, winter exposure may be better interpreted as a modifier of population-level temporal risk rather than as a major determinant of individual stroke risk. Subgroup analyses indicated that the association between winter exposure and ischemic stroke risk was more pronounced in patients without a prior history of ischemic stroke than in those with prior ischemic stroke. Patients in the primary prevention setting may have lower risk awareness and receive fewer medical interventions than do those in the secondary prevention setting, rendering them more susceptible to seasonal fluctuations in risk due to variations in medication adherence and self-management [20, 21]. In contrast, patients with a history of ischemic stroke are generally subject to more intensive secondary prevention strategies, which may mitigate the impact of seasonal risk factors. Although this study did not directly assess medication adherence, the observed interaction may reflect differences in risk awareness, treatment adherence, and preventive care between primary and secondary prevention populations. These findings suggest that winter exposure may be particularly relevant in the primary prevention setting and highlight a potentially important difference in seasonal vulnerability that warrants further investigation.

This study has several strengths. The use of large-scale nationwide data provided stable monthly incidence estimates and regression coefficients, reducing the variability commonly seen in smaller studies. Moreover, misclassification of event timing was minimized by employing a standardized definition that combined dates of hospitalization and diagnosis. Dividing the follow-up period into monthly intervals enabled a clear assessment of the relationship between ischemic stroke occurrence and calendar season, facilitating precise allocation of person-time to seasonal exposure categories.

This study has some limitations. First, although misclassification of diagnoses is an inherent limitation of reimbursement databases, the reliability of outcome identification was enhanced by aligning hospitalization and diagnosis dates and using specific ICD-10 codes [9]. Information on stroke severity and direct validation measures such as positive predictive value was not available in the claims database. Because the outcome definition was based on hospitalization, milder or outpatient-managed cerebrovascular events, particularly TIA, may have been under-ascertained. Second, because weather data (temperature, humidity, pressure) were unavailable, exposure–response relationships and the impact of short-term meteorological variations could not be evaluated [17–19]. Third, the lack of information on anticoagulant adherence, dose adjustment, and use of over-the-counter drugs limited mechanistic interpretation of seasonal behavioral changes. Fourth, although the CHADS₂ score was used as the base model in this study, direct comparison with extended scores, such as CHA₂DS₂-VASc, was not performed [4, 5]. Previous pooled analyses of major Japanese AF registries reported that the additional CHA₂DS₂-VASc components (age 65–74 years, vascular disease, and female sex) were not significant predictors of thromboembolic events in Japanese patients [22]. Nevertheless, whether incorporating these factors would materially change the observed association between winter exposure and ischemic stroke risk remains uncertain. Furthermore, the CHADS₂ score was originally derived from patients with NVAF who were not receiving anticoagulants [4]. However, in modern Japan, it is widely applied to guide initiation of DOAC therapy, making it impractical to restrict the analysis to anticoagulant-naïve patients [11, 12]. Accordingly, we analyzed data from patients regardless of anticoagulant use to evaluate the association between winter exposure and ischemic stroke risk in a real-world NVAF population. Additional adjustment for DOAC use in the sensitivity analysis did not materially change the observed association, although residual confounding related to treatment adherence, dose adjustment, and other unmeasured aspects of preventive care cannot be excluded. Fifth, competing risks, particularly death, were not explicitly accounted for and may have affected the estimated association between winter exposure and ischemic stroke risk. Sixth, the median follow-up period was relatively short, which may have limited the assessment of longer-term temporal variation in ischemic stroke risk. Seventh, because the database included only individuals captured in the DeSC claims database [9], the findings may not be fully generalizable to all patients with NVAF in Japan. Finally, the influence of unmeasured confounders cannot be completely ruled out, and statistically significant differences in large datasets may not always correspond to clinical relevance.

Integrating claims data with high-resolution weather information could clarify the effects of short-term exposures, including temperature thresholds, barometric pressure changes, sudden temperature fluctuations, and air pollution indicators, such as PM2.5 and ozone [16–19, 23, 24]. Because the binary winter versus non-winter classification may simplify continuous temporal variation, future studies using detailed meteorological data and more flexible temporal modeling approaches may provide additional insight beyond static seasonal indicators. The observed interaction between winter exposure and prior ischemic stroke status also warrants further investigation to clarify the mechanisms underlying differential seasonal vulnerability. Finally, examining contextual factors such as prescription gaps, frequency of clinic visits, and local climate and housing conditions may help clarify factors associated with seasonal variation in ischemic stroke risk.

## Conclusion

Overall, our study demonstrated a consistent association between winter exposure and ischemic stroke risk in patients with NVAF. Winter exposure appeared to function primarily as a population-level seasonal modifier of ischemic stroke risk rather than as a factor that substantially improves individual-level risk prediction. The stronger association observed among patients without a prior history of ischemic stroke suggests greater seasonal vulnerability in the primary prevention setting and warrants further investigation. Considering seasonal context in risk awareness and population-level risk communication may help inform the interpretation of ischemic stroke risk in patients with NVAF.

## Supplementary Information

Below is the link to the electronic supplementary material.

Supplementary Material 1.

Supplementary Material 2.

## Data Availability

The data that support the findings of this study are available from DeSC Healthcare, Inc., but restrictions apply to the availability of these data, which were used under license for the current study, and so are not publicly available.

## Abbreviations

AF: Atrial fibrillation
NVAF: Nonvalvular atrial fibrillation
CHADS₂: Congestive heart failure, hypertension, age ≥ 75 years, diabetes mellitus, and prior stroke or transient ischemic attack
ICD-10: International Statistical Classification of Diseases and Related Health Problems, 10th Revision
ATC: Anatomical Therapeutic Chemical classification system
IS: Ischemic stroke
TIA: Transient ischemic attack
DOAC: Direct oral anticoagulant
IR: Incidence rate
IRR: Incidence rate ratio
PY: Person-years
CI: Confidence interval
IQR: Interquartile range
AIC: Akaike information criterion
AUC: Area under the receiver operating characteristic curve
ROC: Receiver operating characteristic
df: Degrees of freedom
PM2.5: Particulate matter with aerodynamic diameter ≤ 2.5 μm
