# Supplementary material for: Association of winter exposure with ischemic stroke risk in nonvalvular atrial fibrillation within a CHADS₂-based risk framework: a nationwide Japanese administrative claims database study
Source: Egypt Heart J. 2026 Jul 16;78:54. doi: 10.1186/s43044-026-00766-z (PMC13376319; doi:10.1186/s43044-026-00766-z)
Supplement: Supplementary file 1 — Supplementary Material 1. [file 43044_2026_766_MOESM1_ESM.docx]

**Additional files**

**Additional file 1.** Definitions of atrial fibrillation (AF) cohort entry and exclusion criteria

| **Category** | **ICD-10 codes** | **Local disease codes** | **Procedure / Material codes** | **Notes** |
| --- | --- | --- | --- | --- |
| Atrial fibrillation (cohort entry) | I48.x | Additional file 3 | – | Index date = first AF diagnosis |
| Exclusion –  Rheumatic/valvular AF | I05.0–I05.2  I05.8–I05.9 |  | – | Excluded if no documented DOAC use (dabigatran, rivaroxaban, apixaban, edoxaban) |
| Exclusion –  Mechanical valve replacement | - |  | Special designated medical material code (Japan): 727420000 (mechanical valve) | Prosthetic valve carriers excluded |

AF was defined using ICD-10 code I48.x. Patients with rheumatic or valvular AF (I05.0–I05.2, I05.8–I05.9) without documented DOAC use and those with prosthetic mechanical valves were excluded.

Corresponding local disease codes are provided in Additional file 3 to ensure reproducibility.

DOAC: direct oral anticoagulant; AF, atrial fibrillation

**Additional file 2.** Definitions of outcomes and covariates

| **Disease** | **ICD-10 codes** | **Local disease codes** | **Medication codes (actcode; mapped ATC)** | **Operational notes** |
| --- | --- | --- | --- | --- |
| **Ischemic stroke** | I63.0, I63.1, I63.2, I63.3, I63.4, I63.5, I63.6, I63.8, I63.9 | Additional file 3 | - | Used for outcome ascertainment as the principal diagnosis during hospitalization. For CHADS₂ “S” (history prior to the AF index), events were identified from diagnosis records and were not limited to inpatient principal diagnoses. Codes I60–I62 (hemorrhagic) and I65–I66 (without infarction) were excluded. |
| **Transient ischemic attack (TIA)** | G45.0, G45.1, G45.8, G45.9, H34.0 |  | - | Used for outcome ascertainment as the principal diagnosis during hospitalization. For CHADS₂ “S” (history prior to the AF index), events were identified from diagnosis records and were not limited to inpatient principal diagnoses. Retinal TIA was defined using H34.0. Code G45.3 was not used, and G45.2 is not defined in the Japanese adaptation of ICD-10 applied in this dataset. |
| **Hypertension** | I10, I11.0, I11.9, I12.0, I12.9, I13.9, I15.0, I15.1, I15.2, I15.9 |  | actcode for antihypertensives; ATC C02/C03/C07/C08/C09 | Defined using both diagnostic and pharmacologic criteria, requiring the relevant ICD-10 diagnosis code and ≥1 pharmacy claim within the baseline window. |
| **Diabetes mellitus** | E10–E14 |  | actcode for antidiabetics; ATC A10A/A10B | Defined using both diagnostic and pharmacologic criteria, requiring the relevant ICD-10 diagnosis code and ≥1 pharmacy claim within the baseline window. |
| **Heart failure** | I11.0, I50.0, I50.1, I50.9 |  | – | Used for CHADS₂ “C”. In this study, it was defined as hospitalization within 3 months before the AF index date, as stated in the Methods section. |

Outcomes included ischemic stroke (I63.x) and transient ischemic attack (TIA; G45.0/1/8/9, H34.0). Covariates included hypertension, diabetes mellitus, and heart failure, defined using ICD-10 codes and medication-based algorithms. For CHADS₂ scoring, stroke/TIA history was identified from diagnosis records prior to the AF index, while hypertension and diabetes were defined using both relevant ICD-10 diagnosis codes and at least one pharmacy claim within the baseline window. Heart failure was defined by hospitalization within 3 months prior to AF index. Local disease codes corresponding to each ICD-10 definition are provided in Additional file 3 to ensure reproducibility.

**Additional file 3.**

English-translated disease names, Japanese local disease codes, and corresponding ICD-10 codes used for identifying comorbidities and clinical conditions in the claims-based NVAF cohort (provided as an Excel file).

**Additional file 4.** Sensitivity analyses evaluating the robustness of the association between winter exposure and ischemic stroke risk among patients with nonvalvular atrial fibrillation

| Sensitivity analysis | IRR (95% CI) |
| --- | --- |
| Primary analysis | 1.06 (1.03–1.09) |
| Additional adjustment for sex, DOAC use, geographic region, and calendar year | 1.08 (1.05–1.12) |
| Excluding transient ischemic attack events | 1.08 (1.05–1.11) |

CI, confidence interval; DOAC, direct oral anticoagulant; IRR, incidence rate ratio; TIA, transient ischemic attack

The primary analysis was adjusted for CHADS₂ components: age ≥75 years, hypertension, diabetes mellitus, heart failure hospitalization, and prior ischemic stroke, including TIA. The additional adjustment model further included sex, DOAC use, geographic region, and calendar year. The transient ischemic attack-excluded analysis used the same adjustment variables as the primary analysis but excluded transient ischemic attack events from the outcome definition.

**Additional file 5.** Monthly event counts, person-years, and incidence rates of ischemic stroke

| Month | Number of events | Person-years | Incidence rate per 1,000  person-years |
| --- | --- | --- | --- |
| January | 2295 | 64113 | 35.8 |
| February | 2131 | 65674 | 32.5 |
| March | 2322 | 67522 | 34.4 |
| April | 1746 | 53594 | 32.6 |
| May | 1823 | 55399 | 32.9 |
| June | 1889 | 57313 | 33.0 |
| July | 1974 | 59292 | 33.3 |
| August | 2004 | 61040 | 32.8 |
| September | 1829 | 61232 | 29.9 |
| October | 2103 | 63201 | 33.3 |
| November | 2113 | 61972 | 34.1 |
| December | 2347 | 63775 | 36.8 |

IR, incidence rate; PY, person-years.

**Additional file 6.** Receiver operating characteristic (ROC) curves for ischemic stroke prediction


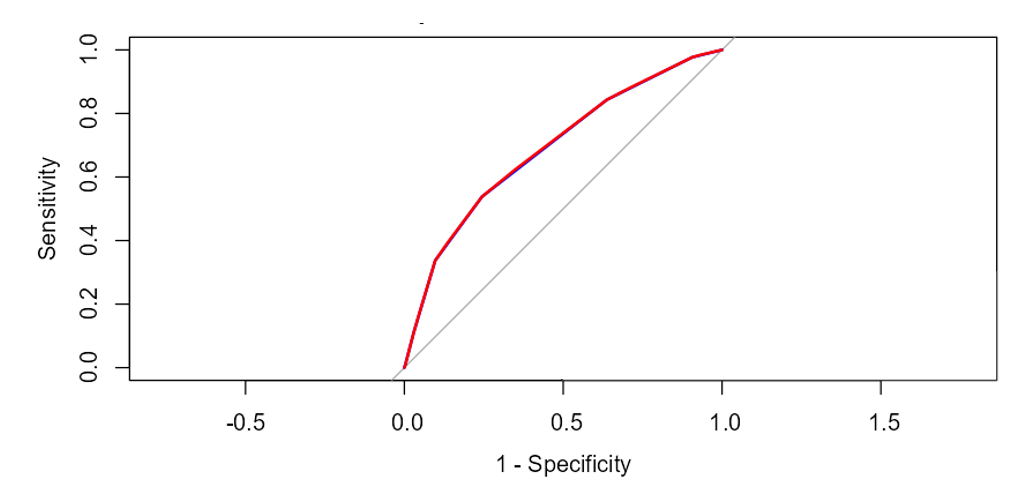


ROC curves are shown for the CHADS₂-only model (blue) and the CHADS₂ plus winter exposure model (red). The area under the curve values are 0.690 and 0.691, respectively (ΔAUC = 0.001).
